# Empirical comparison and analysis of machine learning-based predictors for predicting and analyzing of thermophilic proteins

**DOI:** 10.17179/excli2022-4723

**Published:** 2022-03-02

**Authors:** Phasit Charoenkwan, Nalini Schaduangrat, Md Mehedi Hasan, Mohammad Ali Moni, Pietro Lió, Watshara Shoombuatong

**Affiliations:** 1 Modern Management and Information Technology, College of Arts, Media and Technology, Chiang Mai University, Chiang Mai, Thailand, 50200; 2 Center of Data Mining and Biomedical Informatics, Faculty of Medical Technology, Mahidol University, Bangkok, Thailand, 10700; 3 Tulane Center for Biomedical Informatics and Genomics, Division of Biomedical Informatics and Genomics, John W. Deming Department of Medicine, School of Medicine, Tulane University, New Orleans, LA 70112, USA; 4 School of Health and Rehabilitation Sciences, Faculty of Health and Behavioural Sciences, the University of Queensland, St Lucia, QLD 4072, Australia; 5 Department of Computer Science and Technology, University of Cambridge, Cambridge, CB3 0FD, UK

**Keywords:** thermophilic protein, bioinformatics, classification, machine learning, feature representation, feature selection

## Abstract

Thermophilic proteins (TPPs) are critical for basic research and in the food industry due to their ability to maintain a thermodynamically stable fold at extremely high temperatures. Thus, the expeditious identification of novel TPPs through computational models from protein sequences is very desirable. Over the last few decades, a number of computational methods, especially machine learning (ML)-based methods, for *in silico* prediction of TPPs have been developed. Therefore, it is desirable to revisit these methods and summarize their advantages and disadvantages in order to further develop new computational approaches to achieve more accurate and improved prediction of TPPs. With this goal in mind, we comprehensively investigate a large collection of fourteen state-of-the-art TPP predictors in terms of their dataset size, feature encoding schemes, feature selection strategies, ML algorithms, evaluation strategies and web server/software usability. To the best of our knowledge, this article represents the first comprehensive review on the development of ML-based methods for in *silico* prediction of TPPs. Among these TPP predictors, they can be classified into two groups according to the interpretability of ML algorithms employed (i.e., computational black-box methods and computational white-box methods). In order to perform the comparative analysis, we conducted a comparative study on several currently available TPP predictors based on two benchmark datasets. Finally, we provide future perspectives for the design and development of new computational models for TPP prediction. We hope that this comprehensive review will facilitate researchers in selecting an appropriate TPP predictor that is the most suitable one to deal with their purposes and provide useful perspectives for the development of more effective and accurate TPP predictors.

## Introduction

Proteins perform varied functions in the body such as enzyme catalysis, ion and molecular transport, antibody production, and cellular/physiological activity regulation and thus, are considered as one of the most important biological macromolecules. The three-dimensional structure of proteins heavily influences their functioning (Burley et al., 2017[5]). Furthermore, structure-based drug design heavily relies on complex protein inter-residue interactions such as mechanisms of protein folding, rates of folding and unfolding, stability of protein structure, stability upon mutation, recognition mechanisms of protein-protein, protein-nucleic acid and protein-ligand complexes (Gromiha, 2010[22]; Gromiha et al., 2019[24]). Moreover, the critical role of Thermophilic proteins (TPPs) in biotechnology and chemical processing have already been established (Haki and Rakshit, 2003[28]). TPPs maintain their stability at high temperatures (80-100 °C) as well as in the environmental temperatures of the host organism (Gaucher et al., 2008[21]; Gromiha et al., 1999[25]). Additionally, the stability of TPPs depends upon a variety of amino acid properties such as shape, hydration energy change (Gibbs function) in native proteins, dipeptide composition, amino acid residue contacts, ion pair numbers, hydrogen bonds, packing, and aromatic clusters (Gromiha et al., 1999[25]; Pica and Graziano, 2016[46]). Out of all the aforementioned properties, TPP stability relies mostly on hydrophobicity as the most important feature, followed by ion pairs and hydrogen bonds (Gromiha and Nagarajan, 2013[23]). Therefore, in order to design proteins for specific medical or industrial applications, a thorough understanding of the molecular basis of protein thermostability is critical (Gromiha et al., 2019[24]). Furthermore, the ease of TPP purification and their ability to withstand long periods of industrial conditions comes from their natural resistance to denaturation by chemical compounds (i.e., detergents, surfactants, oxidizing agents, and proteases) (Diaz et al., 2011[17]; Habbeche et al., 2014[27]; Huang et al., 2012[32]). Of note, survival of therapeutic proteins in blood is extended with higher thermostability (Narasimhan et al., 2010[45]). Several advantages of TPPs include reduced contamination, mixes easily with low viscous agents while maintaining a high mass transfer rate as well as achieving greater solubility of products and substrates (Vieille and Zeikus, 2001[55]). Furthermore, TPPs are advantageous in high-temperature pelleting processes (Rodriguez et al., 2000[48]) as well as in the isomerization of glucose through endothermic reactions to generate high fructose syrups (Xu et al., 2014[60]). Although experimental methods are the gold standard in verifying thermostability of proteins, these methods are usually labor-intensive, time-consuming and expensive. Thus, the rapid and accurate identification of TPPs from a large collection of proteins is highly advantageous and cost-effective. 

**Figure 1:**
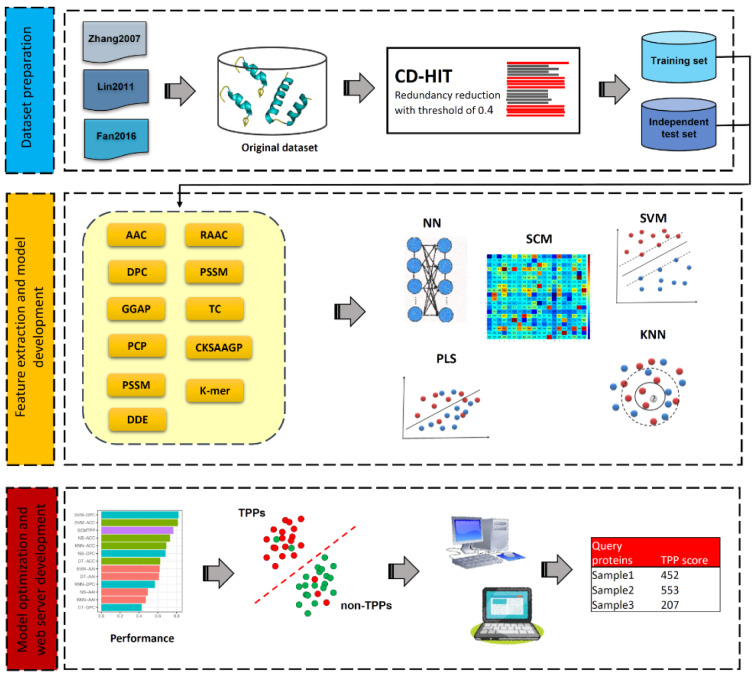
The overall framework of TPP predictors using machine learning methods. The 1st phase is dataset preparation to form training and independent datasets. The 2nd phase is feature extraction and feature optimization. The 3rd phase is to train and evaluate a prediction model. The independent dataset is used to validate the effectiveness and robustness of the prediction model. Finally, the optimal prediction model is selected to establish a web server.

Over the last few decades, a number of computational methods, especially machine learning (ML)-based methods, for *in silico* prediction of TPPs have been developed. The development of all these existing TPP predictors involves three main phases as summarized in Figure 1(Fig. 1). The 1st phase is dataset preparation to form training and independent datasets. The 2nd phase is feature extraction and feature optimization. The 3rd phase is to train and evaluate a prediction model. The independent dataset is used to validate the effectiveness and robustness of the prediction model. Finally, the optimal prediction model is selected to establish a web server. We categorize the existing TPP predictors as listed in Table 1(Tab. 1) (References in Table 1: Charoenkwan et al., 2022[10]; Fan et al., 2016[18]; Feng et al., 2020[19]; Gromiha and Suresh, 2008[26]; Li et al., 2019[37]; Lin and Chen, 2011[39]; Nakariyakul et al., 2012[44]; Tang et al., 2017[54]; Wang and Li, 2014[57]; Wang et al., 2011[56]; Wu et al., 2009[58]; Zhang and Fang, 2006[61], 2007[62]; Zuo et al., 2013[66]) into two groups according to the interpretability of the ML algorithms employed. The first group are the computational black-box methods, and there are nine out of fourteen existing TPP predictors (i.e., Gromiha et al.'s method (Gromiha and Suresh, 2008[26]), ThermoPred (Lin and Chen, 2011[39]), Wang et al.'s method (2011[56]), Nakariyakul et al.'s method (2012[44]), KNN-ID (Zuo et al., 2013[66]), PSSM400_pKa (Fan et al., 2016[18]), Tang et al.'s method (2017[54]), Li et al.'s method (2019[37]) and Feng et al.'s method (2020[19])) in this group. 

The second group are the computational white-box methods, and there are five out of fourteen existing TPP predictors (i.e., Zhang et al.'s method (Zhang and Fang, 2006[61]), LogitBoost (Zhang and Fang, 2007[62]), Wu et al.'s method (2009[58]), GA-MLR (Wang and Li, 2014[57]) and SCMTPP (Charoenkwan et al., 2022[10])) in this group.

To the best of our knowledge, this article represents the first comprehensive review on the development of ML-based methods for *in silico* prediction of TPPs. In this study, our aim is to conduct an empirical comparison and analysis of fourteen existing TPP predictors in terms of multiple perspectives, including their feature encoding schemes, feature selection strategies, ML algorithms, evaluation strategies and web server/software usability as summarized in Table 1(Tab. 1). First, we reviewed available training and independent datasets employed for developing the current TPPs predictors. The detailed information of these datasets are provided in Table 2(Tab. 2) (References in Table 2: Charoenkwan et al., 2022[10]; Fan et al., 2016[18]; Feng et al., 2020[19]; Gromiha and Suresh, 2008[26]; Li et al., 2019[37]; Lin and Chen, 2011[39]; Nakariyakul et al., 2012[44]; Tang et al., 2017[54]; Wang and Li, 2014[57]; Wang et al., 2011[56]; Wu et al., 2009[58]; Zhang and Fang, 2006[61], 2007[62]; Zuo et al., 2013[66]). Second, the performance of various TPP predictors on two benchmark datasets (i.e., the Gromiha2007 (Gromiha and Suresh, 2008[26]) and Lin2011 (Lin and Chen, 2011[39])) and two independent datasets (i.e., the Zhang2007 (Zhang and Fang, 2006[61]) and Charoenkwan2021 (Charoenkwan et al., 2022[10])) were compared and discussed. Our comparative results demonstrate that ThermoPred outperformed the competing TPP predictors in terms of both predictive performance and community utility, while SCMTPP outperformed the competing TPP predictors in terms of high interpretability and simplicity. Finally, we discuss the advantages and disadvantages of the current TPP predictors and provided future perspectives for the design and development of new computational models for TPP prediction.

## Materials and Methods

### Framework of TPP prediction using machine learning-based approaches

The overall framework of TPP predictors using machine learning methods involves three main phases as summarized in Figure 1(Fig. 1). The 1st phase is to prepare the high-quality dataset to generate training (for cross-validation and parameter optimization purposes) and independent (for assessing and validating the transferability and reliability) datasets. The 2nd phase is feature extraction and feature optimization. Feature extraction works to represent each protein sequence to capture the key information of TPPs and non-TPPs. The detailed information of feature encodings employed is recorded in Table 1(Tab. 1). Since each protein sequence is represented as a high dimensional feature vector, it is well-known that the feature optimization step might help to exclude irrelevant/noisy features and lead to the improved performance of the trained model. Thus, the 3rd phase is to train and evaluate the prediction model. The independent dataset is used to validate the effectiveness and robustness of the prediction model. Finally, the optimal prediction model is selected to establish a web server. The details of web server availability and usability for TPP prediction is recorded in Table 1(Tab. 1).

### Datasets

Detailed information of all the training and independent datasets used for developing the existing methods are recorded in Table 2(Tab. 2). Among these datasets, the Gromiha2007 (Gromiha and Suresh, 2008[26]) (used for developing Gromiha et al.'s method (Gromiha and Suresh, 2008[26]), KNN-ID (Zuo et al., 2013[66]), and PSSM400_pKa (Fan et al., 2016[18])) and Lin2011 (Lin and Chen, 2011[39]) (used for developing ThermoPred (Lin and Chen, 2011[39]), Nakariyakul et al.'s method (Nakariyakul et al., 2012[44]), GA-MLR (Wang and Li, 2014[57]), Tang et al.'s method (Tang et al., 2017[54]), Li et al.'s method (Li et al., 2019[37]) and Feng et al.'s method (Feng et al., 2020[19])) datasets were two well-known training datasets used for developing almost all of the existing methods. As described in an article (Gromiha and Suresh, 2008[26]), the training dataset of Gromiha2007 were directly derived from the Zhang2007 dataset (3521 TPPs and 4895 non-TPPs) and TPPs and non-TPPs with more than 40 % sequence identity were then excluded using the CD-HIT program. Finally, the training dataset of the Gromiha2007 dataset contained 1609 TPPs and 3075 non-TPPs. In case of the Lin2011 dataset (915 TPPs and 793 non-TPPs), Lin et al. collected TPPs and non-TPPs from 136 prokaryotic organisms extracted from the Universal Protein Resource (UniProt). Unfortunately, only the Lin2011 dataset can be accessed at http://lin-group.cn/server/ThermoPredv1. Recently, our group constructed an up-to-date dataset from several previous studies (Fan et al., 2016[18]; Lin and Chen, 2011[39]; Zhang and Fang, 2006[61]) consisting of 6579 TPPs. After excluding redundant sequences using the CD-HIT program, 1823 TPPs and 3124 non-TPPs were obtained (called the Charoenkwan2021 dataset). The Charoenkwan2021 dataset can be downloaded at http://pmlabstack.pythonanywhere.com/SCMTPP.

### State-of-the-art computational approaches for TPP prediction

More than ten ML-based approaches have been developed for TPP prediction. These approaches were developed using a variety of aspects, including the benchmark datasets, feature descriptors, feature section methods and ML algorithms, etc. In Table 1(Tab. 1), we summarize 13 existing sequence-based TPP predictors along with their employed feature encoding schemes, ML algorithms and evaluation strategies. Most TPP predictors were trained and constructed in a five-step manner, which involves data preparation, feature extraction, feature selection, model optimization and development and web server construction. These existing sequence-based TPP predictors are categorized into two classes according to the interpretability of ML algorithms employed (Kurgan et al., 2009[34]; Liang et al., 2021[38]; Shoombuatong et al., 2017[50]), which are described in detail below.

### Performance evaluation and evaluation strategy

The predictive performance of our proposed model, baseline models and the two state-of-the-art methods is evaluated and compared using five common performance measures as follows: accuracy (ACC), sensitivity (Sn), specificity (Sp), Matthew's Correlation Coefficient (MCC) and area under the receiver-operating curves (AUC) (Azadpour et al., 2014[2]; Charoenkwan et al., 2021[9]). These performance measures are described by the following equations:


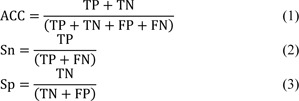


where TP, TN, FP and FN represent the number of true positives, true negatives, false positive and false negatives, respectively (Basith et al., 2020[4]; Shoombuatong et al., 2017[50]; Su et al., 2020[51]). It is well-known that Sn and Sp measure the predictive ability for two classes: positive and the negative, respectively. ACC, MCC and AUC evaluate the overall performance of the predictive model. 

## State-of-the-Art Computational Approaches for TPP Prediction

More than ten ML-based approaches have been developed for TPP prediction. These approaches were developed using a variety of aspects, including the benchmark datasets, feature descriptors, feature section methods and ML algorithms, etc. In Table 1(Tab. 1), we summarize 13 existing sequence-based TPP predictors along with their employed feature encoding schemes, ML algorithms and evaluation strategies. Most TPP predictors were trained and constructed in a five-step manner, which involves data preparation, feature extraction, feature selection, model optimization and development and web server construction. These existing sequence-based TPP predictors are categorized into two classes according to the interpretability of ML algorithms employed (Kurgan et al., 2009[34]; Liang et al., 2021[38]; Shoombuatong et al., 2017[50]), which are described in detail below.

### Prediction methods based on computational black-box methods

Among several ML algorithms used in this aspect, KNN (Zuo et al., 2013[66]), NN (Gromiha and Suresh, 2008[26]) and SVM (Fan et al., 2016[18]; Feng et al., 2020[19]; Lin and Chen, 2011[39]; Nakariyakul et al., 2012[44]; Tang et al., 2017[54]; Wang et al., 2011[56]) are known as Black-box approaches. Kurgan et al. (2009[34]) described black-box methods as ML methods that cannot directly determine which features provide essential contribution to the prediction performance. As can be seen from Table 1(Tab. 1), there are 6 out of 14 existing sequence-based predictors that were trained and developed by using SVM method (i.e., ThermoPred (Lin and Chen, 2011[39]), Wang et al.'s method (2011[56]), Nakariyakul et al.'s method (2012[44]), PSSM400_pKa (Fan et al., 2016[18]), Tang et al.'s method (2017[54]), Li et al.'s method (2019[37]) and Feng et al.'s method (2020[19])). SVM has been successfully applied to solve variant research questions in computational biology and bioinformatics. The basic idea of SVM is to map the given input features into a high-dimensional space using kernel functions and find a maximum margin hyperplane that can separate positive samples from negative samples with a minimal misclassification rate (Chen et al., 2016[15]; Manavalan and Lee, 2017[43]). There are three well-known and commonly used kernel functions in SVM, including gaussian, polynomial and radial basis function (RBF). Particularly, the development of SVM models involves the optimization of two critical parameters, i.e. C and γ represent the regularization parameter and kernel parameter, respectively (Arif et al., 2020[1]; Charoenkwan et al., 2020[13], 2021[9]). 

In 2011, Lin et al. developed the first SVM-based approach for identifying TPPs (called ThermoPred) by using the Lin2011 dataset containing 915 TPPs and 793 non-TPPs. ThermoPred was trained with two feature descriptors (i.e., AAC and GGAP). In order to improve the predictive performance of ThermoPred, ANOVA technique was used to determine informative g-gap dipeptides. As a result, the informative g-gap dipeptides consisted of EE, KE, EI, I-K, I-E, E-K, E-E, K-E, Q-A and E---K, where - represents the gap of residues. In addition, the Sn, Sp and ACC of ThermoPred were 82.4 %, 93.0 % and 89.4 %, respectively, based on 5-fold cross-validation test. In the same year, Wang et al. developed another SVM-based approach for identifying TPPs. Their SVM-based approach was trained with three types of feature descriptors, including AAC, CTD and PCP. In addition, three feature selection methods (i.e., filter method, relief algorithm and genetic algorithm) were employed and used to determine informative features. Amongst these three feature selection methods, the highest ACC of 95.93 % was achieved by using genetic algorithm. The informative features derived from the genetic algorithm contained A, Q, I, K, F, Y, AA, AD, AQ, AS, RI, RK, DA, DQ, EE, EK, GQ, GI, GS, IN, IV, LY, MI, PA, SA, SQ, TI, YV, VY and CTD10. Recently, Feng et al. proposed another SVM-based approach trained with four feature descriptors (i.e., ACC, DPC, PCP and RAAC) (Feng et al., 2020[19]). Then, principal component analysis was used to reduce irrelevant features and the final feature set contained 12 informative features. 

In case of other ML algorithms employed, Gromiha et al. (Gromiha and Suresh, 2008[26]) and Zuo et al. (2013[66]) applied NN-based (called Gromiha et al.'s method) and KNN-based (called KNN-ID) models, respectively, trained with AAC to develop TPP predictors. For the 10-fold cross-validation results, ACC, Sn, and Sp of Gromiha et al.'s method (Gromiha and Suresh, 2008[26]) were 89.00 %, 83.30 % and 92.00 %, respectively, while ACC, Sn, and Sp of KNN-ID (Zuo et al., 2013[66]) were 90.66 %, 88.37 % and 92.24 %, respectively. To improve the accurate prediction of TPPs, Li et al. (2019[37]) employed an ensemble strategy. To the best of the authors' knowledge, there is only one TPP predictor in existence that was constructed by using the ensemble strategy. Several previous studies have indicated that ensemble-based models are effective to provide improved performance over single-based models (Basith et al., 2022[3]; Hasan et al., 2020[30]; Kabir et al., 2022[33]; Liang et al., 2021[38]; Manavalan et al., 2019[41]; Rao et al., 2020[47]). Their ensemble model provided a cross-validation ACC of 93.03 %.

### Prediction methods based on computational white-box methods

Unlike black-box methods, white-box models are able to determine which features provide essential contribution to the prediction performance, such as DT (Wu et al., 2009[58]), MLR (Wang and Li, 2014[57]), PLS (Zhang and Fang, 2006[61]) and SCM (Charoenkwan et al., 2022[10]). Amongst several white-box methods, SCM has been indicated to achieve comparable performance to those of black-box methods, such as NN and SVM (Charoenkwan et al., 2013[14], 2021[8][11], 2022[10]). Huang et al. first introduced the original SCM method (Huang et al., 2012[31]), while Charoenkwan et al. developed an improved version by integrating both global and local sequence information (Charoenkwan et al., 2021[8]). The contribution of the SCM method is summarized in the following three aspects. First, the SCM method can discriminate positives from negatives by using only a single threshold value, emphasizing its ease-of-use and interpretability. Second, since the SCM method is known as a single feature-based model, indicating that this method could achieve better computational efficiency as compared to complex methods, such as SVM and ensemble approaches. Third, the SCM-derived propensity scores of 20 amino acids and 400 dipeptides are useful for characterizing and analyzing various functions of proteins and peptides. 

In 2006, Zhang et al. proposed the first sequence-based predictor based on PLS algorithm for identifying TPPs based on 76 TPPs and 76 non-TPPs. Their method had the highest ACC for TPPs and non-TPPs prediction, which was 75 % and 85 %, respectively. Most recently, our group developed a new sequence-based predictor (called SCMTPP) for identifying and characterizing TPPs using estimated propensity scores of dipeptides. Furthermore, we established an up-to-date and high-quality dataset containing 1853 TPPs and 3233 non-TPPs from several published literatures. SCMTPP was developed using SCM method in conjunction with GGAP. SCMTPP based on the propensity scores of GGAP (g=0) was beneficial for TPP prediction with ACC of 88.30 %, MCC of 0.766 and AUC of 0.926 as evaluated by 10-fold cross-validation test. When compared with popular ML methods (i.e., DT, KNN and naive Bayes (NB)) on the training dataset, it could be noticed that SCMTPP outperformed those of DT-based, KNN-based and NB-based classifiers. Remarkably, SCMTPP's ACC was >7.05 %, >3.78 % and >1.86 % higher than DT-based, KNN-based and NB-based models, respectively.

## Results and Discussion

### Comparative results on 5-fold and 10-fold cross-validation tests using the Gromiha2007 and Lin2011 datasets

In this section, we evaluated and compared the performance of different TPP predictors in terms of ACC, Sn and Sp using the two benchmark datasets (i.e., Gromiha2007 and Lin2011). To be specific, the Gromiha2007 dataset was used to evaluate five out of the fourteen existing TPP predictors (i.e., Gromiha et al.'s method, Wu et al.'s method, ThermoPred, KNN-ID, and PSSM400_pKa), while the Lin2011 dataset was used to evalute the six out of the fourteen existing TPP predictors (i.e., Gromiha et al.'s method, ThermoPred, Nakariyakul et al.'s method, GA-MLR, Tang et al.'s method and Feng et al.'s method).

**Figure 2:**
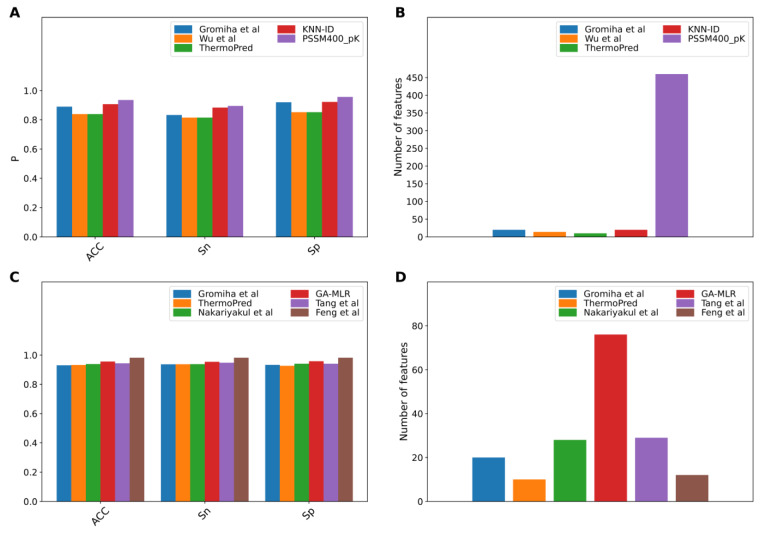
Performance comparison of existing TPP predictors on the Gromiha2007 (A-B) and Lin2011 (C-D) datasets. (A, C) represent the performance in terms of ACC, Sn and Sp. (B, D) represent the feature number used in existing TPP predictors.

The performance evaluation results of these two benchmark datasets are summarized in Figures 2(Fig. 2) and Tables 3(Tab. 3)-4(Tab. 4). As seen in Figure 2(Fig. 2) and Table 3(Tab. 3) (Reference in Table 3: Fan et al., 2016[18]), ThermoPred outperformed the four competing TPP predictors (i.e., Gromiha et al.'s method, Wu et al.'s method, KNN-ID, and PSSM400_pKa) in terms of model complexity based on the Gromiha2007 dataset. Taking into consideration the cross-validation performance, PSSM400_pKa achieved the best performance in terms of ACC (93.53 %), Sn (89.50 %) and Sp (95.64 %) (Figure 2A(Fig. 2)). In the meanwhile, ThermoPred achieved the second-best performance in terms of ACC (93.53 %) and Sp (95.64 %). In terms of model complexity, ThermoPred (10D) performed better than PSSM400_pKa (460D) (Figure 2B(Fig. 2)). One of the major limitations of PSSM400_pKa was that there was no web server provided for this study. Therefore, its utility is limited to experimental scientists. In case of the Lin2011 dataset, Table 4(Tab. 4) (References in Table 4: Nakariyakul et al., 2012[44]; Tang et al., 2017[54]; Wang and Li, 2014[57]) shows that ThermoPred still outperformed competing TPP predictors (i.e., Gromiha et al.'s method, Nakariyakul et al.'s method, GA-MLR, Tang et al.'s method and Feng et al.'s method) in terms of model complexity (Figure 2C-2D(Fig. 2)). It could be noticed that Feng et al.'s method achieved the best performance in terms of ACC (98.20 %), Sn (98.20 %) and Sp (98.20 %), while GA-MLR achieved the second-best performance in terms of the three performance metrics (i.e., ACC (95.61 %), Sn (95.41 %) and Sp (95.84 %)).

From Tables 3(Tab. 3)-4(Tab. 4), the existing TPP predictors were trained and optimized based on the two benchmark datasets and several observations can be made. First, PSSM400_pKa and Feng et al.'s method achieved a better performance compared with the competing TPP predictors on the Gromiha2007 and Lin2011 datasets, respectively. However, their usage and utility is quite limited to experimental scientists. Meanwhile, among several TPP predictors developed using the two benchmark datasets, only ThermoPred was implemented as a web server for the prediction of TPPs. Second, there were two TPP predictors that were evaluated on the two benchmark datasets (i.e., Gromiha et al.'s method and ThermoPred). ThermoPred achieved a competitive performance on the Gromiha2007 and Lin2011 datasets when compared with PSSM400_pKa and Feng et al.'s method, respectively. Altogether, these comparative results indicated that ThermoPred could outperform the competing TPP predictors in terms of both predictive performance and community utility.

### Comparative results on the independent test using the Zhang2007 and Charoenkwan2021 datasets

Prediction models having a high cross-validation performance might not perform well on the independent datasets (Charoenkwan et al., 2021[6][7]; Kabir et al., 2022[33]; Shoombuatong et al., 2017[50]). Thus, in this section, we conducted an independent test to validate and assess the generalization ability of the existing TPP predictors. As can be seen from Table 2(Tab. 2), different TPP predictors were validated using different independent datasets.

Moreover, among variant independent datasets, there were three well-known independent datasets derived from the Zhang2006 (76 TPPs and 81 non-TPPs) (Zhang and Fang, 2006[61]), Zhang2007 (382 TPPs and 325 non-TPPs) (Zhang and Fang, 2007[62]) and Charoenkwan2021 (371 TPPs and 371 non-TPPs) (Charoenkwan et al., 2022[10]) datasets. For convenience of discussion, we denote these three independent datasets as Zhang2006TS, Zhang2007TS and Charoenkwan2021TS, respectively.

For the Zhang2006TS dataset, it was first used as the training dataset to develop Zhang et al.'s method. In 2012, Nakariyakul et al., first applied the Zhang2006TS dataset to evaluate their model. In the meanwhile, the Zhang2006TS dataset was used to assess the performance of KNN-ID, GA-MLR and PSSM400_pKa. It should be noted that the Lin2011 (915 TPPs and 793 non-TPPs) and Gromiha2007 (1609 TPPs and 3075 non-TPPs) datasets were utilized to train and optimize Nakariyakul et al.'s method, GA-MLR and KNN-ID and PSSM400_pKa, respectively (Table 2(Tab. 2)). As can be seen from Table 5(Tab. 5) (References in Table 5: Charoenkwan et al., 2022[10]; Fan et al., 2016[18]; Gromiha and Suresh, 2008[26]; Wang and Li, 2014[57]; Zang and Fang, 2006[61]; Zuo et al., 2013[66]), PSSM400_pKa achieved the highest ACC, Sn and Sp of 97.45 %, 97.37 % and 97.53 %, respectively, while the second-best method was KNN-ID in term of ACC. For the Zhang2007TS dataset, it was constructed by Zhang et al (Zhang and Fang, 2007[62]). This dataset was utilized to evaluate the performance of LogitBoost and Gromiha et al.'s method. LogitBoost and Gromiha et al.'s method were trained and optimized using different datasets (Table 2(Tab. 2)). It could be noticed that LogitBoost outperformed Gromiha et al.'s method in terms of ACC (92.08 %) and Sp (91.70 %) (Table 5(Tab. 5)). This might be due to the fact that LogitBoost were trained using larger samples. For the last independent dataset, it was constructed by Charoenkwan et al. (2021[11]). This dataset was utilized to evaluate the performance of ThermoPred and SCMTPP. From Table 5(Tab. 5), it could be observed that SCMTPP achieved a very comparable performance to ThermoPred in terms of ACC, Sp and Sn.

### Characterization of TPPs based on sequence information

As mentioned above, there were five out of fourteen existing TPP predictors consisting of Zhang et al.'s method (Zhang and Fang, 2006[61]), LogitBoost (Zhang and Fang, 2007[62]), Wu et al.'s method (Wu et al., 2009[58]), GA-MLR (Wang and Li, 2014[57]) and SCMTPP (Charoenkwan et al., 2022[10]) considered as the computational white-box methods. Amongst these white-box methods developed for the prediction and analysis of TPPs, SCMTPP as introduced by Charoenkwan et al. (2022[10]), outperformed the competing TPP predictors in terms of high interpretability and simplicity. To be specific, SCMTPP was trained and optimized using an up-to-date dataset containing 1853 TPPs and 3233 non-TPPs. By analysis of the propensity scores of twenty amino acids to be TPPs, Charoenkwan et al., reported that the five top-ranked important amino acids to be TPPs were Glu, Lys, Val, Arg and Ile with propensity scores of 510.18, 480.00, 470.75, 464.08 and 435.65, respectively. On the other hand, the five top-ranked important amino acids to be non-TPPs were Gln, Thr, Ala, Asn and Phe with propensity scores of 255.43, 306.00, 323.63, 332.48 and 351.25, respectively. This group also indicated that the top five informative dipeptides to be TPPs consisted of EE, GW, SG, WS and KY with propensity scores of 1000, 979, 956, 952 and 908, respectively, while the top five informative dipeptides to be non-TPPs consisted of AA, LQ, NM, FW and MQ with propensity scores of 0, 11, 27, 41 and 47, respectively. In addition, SCMTPP was applied to determine informative physicochemical properties (PCPs). Charoenkwan et al. (2021[11]) reported that FUKS010101 (R = 0.616), FUKS010101 (R = 0.523) and FUKS010109 (R = 0.307) were considered as the top three informative PCP used for analyzing TPPs and non-TPPs. Charoenkwan et al.'s analysis showed that the content of hydrophobic amino acids in TPPs was not different from non-TPPs. However, all the results from Charoenkwan et al.'s analysis were derived from primary sequence information, while only selected TPPs and non-TPPs were used to analyze their PCPs. Thus, Charoenkwan et al.'s analysis was limited due to the small size of selected TPPs and non-TPPs used in their studies.

### Web server availability and usability

As seen in Table 1(Tab. 1), there are a total of 14 sequence-based predictors, but only two of them (ThermoPred and SCMTPP) were implemented as web servers for the prediction of TPPs. ThermoPred is an SVM-based predictor trained with the informative g-gap dipeptides consisted of EE, KE, EI, I-K, I-E, E--K, E--E, K--E, Q--A and E---K derived from ANOVA approach. ThermoPred is freely available at http://lin-group.cn/server/ThermoPredv1. On the contrary, SCMTPP was constructed using the propensity scores of GGAP (g=0) and a threshold value of 418, where an unknown protein *P* is predicted as TPP if its TPP score is greater than the threshold value, otherwise this protein is predicted as non-TPP. SCMTPP is freely available at http://pmlabstack.pythonanywhere.com/SCMTPP.

## Prospective Strategies for Improving the Prediction Performance of TPPs

In this section, we discuss the advantages and disadvantages of the current TPPs predictors. In addition, we provided future perspectives for the design and development of new computational models for TPP prediction. Hereafter, four crucial aspects for further improving the performance of TPP predictions are discussed and explored.

First, all the existing TPP predictors were trained using sequence-based features. Among several of the sequence-based features employed, ACC was the most frequently used one (Table 1(Tab. 1)). Numerous previous studies have indicated that sequence-to-vector encodings has been successfully employed for feature extraction in order to facilitate the characterization and analysis of protein, peptide and DNA sequences (Le et al., 2019[36]; Tahir et al., 2020[53]; Xie et al., 2021[59]). Unlike sequence-based features, sequence-to-vector encodings were able to provide better performance in many cases (Charoenkwan et al., 2021[12]; Lv et al., 2021[40]; Shah and Ou, 2021[49]; Zulfiqar et al., 2021[65]). To the best of our knowledge, there is no TPP predictor reported that is trained and optimized using sequence-to-vector encodings. 

Second, there is no DL-based TPP predictor in existence in this aspect. Meanwhile, with a large number of characterized proteins in recent years, the utility of DL techniques has been reported by numerous studies in biological research (Charoenkwan et al., 2021[12]; Lv et al., 2021[40]; Shah and Ou, 2021[49]; Xie et al., 2020[59]; Zulfiqar et al., 2021[65]). Specifically, DL-based methods can extract features from protein, peptide and DNA sequences directly by using natural language processing (NLP) technique without the need of feature encodings. To date, DL-based methods are effective and powerful built-in feature extractors. For instance, our group developed BERT4Bitter, which was a bidirectional encoder representation from transformers (BERT)-based model for the identification of bitter peptides (Charoenkwan et al., 2021[12]). We compared BERT4Bitter with popular ML-based methods developed with ANN, DT, KNN, SVM, ANN, extremely randomized trees (ETree), linear support vector classifier (SVC), logistic regression (LR), naive Bayes (NB), random forest (RF), and extreme gradient boosting (XGB). Specifically, these ML-based methods were trained using five sequence-based features containing AAC, DPC, TC, amino acid index (AAI) and pseudo amino acid composition (PAAC). Remarkably, BERT4Bitter outperformed the comparative ML-based methods in terms of ACC (with an improvement of 2-29 %) and MCC (with improvements of 2-59 %) on the independent dataset. Thus, a DL-based TPP predictor might plausibly achieve improved performance over the fourteen existing TPP predictors.

Third, all the existing TPP predictors were developed by using single ML algorithms to train the model. Thus, their performance is not robust in some cases (Charoenkwan et al., 2021[6]; Kabir et al., 2022[33]; Liang et al., 2021[38]). To date, there is no ensemble-based TPP predictor in existence in this aspect. There are three popular ensemble learning methods (i.e., majority voting, average probability and stacking strategy). Several studies have indicated that the stacking strategy outperformed the other two ensemble learning methods. Unlike the remaining ensemble learning methods, the stacking strategy can automatically explore different baseline models in order to develop a single stable model. For example, our group proposed a stacking ensemble model, namely StackIL6, for accurately identifying IL-6 inducing peptides (Charoenkwan et al., 2021[9]). In StackIL6, we employed twelve different feature descriptors and five popular ML algorithms (i.e., ANN, ETree, LR, SVM and RF) to construct variant baseline models for developing the final stacking ensemble model. Our comparative results showed that StackIL6 achieved the highest performance over its baseline models on the training and independent datasets. Altogether, the performance of TPP prediction might be logically increased by applying the ensemble learning strategy (Basith et al., 2022[3]; Charoenkwan et al., 2020[13]; Hasan et al., 2021[29]; Manavalan et al., 2019[41][42]; Su et al., 2020[52]; Zhang and Zou, 2020[63]).

Finally, it is well-known that the advantage of a web server is to quickly identify potential TPP candidates from large-scale proteins and provide the prediction without the need to develop an in-house prediction model (Dao et al., 2019[16]; Feng et al., 2019[20]; Lai et al., 2019[35]; Zhu et al., 2019[64]). However, most of these predictors were not developed as web servers, with the exception of ThermoPred and SCMTPP. Although PSSM400_pKa outperformed other TPP predictors, its utility is limited to experimental scientists. Overall, in terms of both predictive performance and community utility, ThermoPred and SCMTPP outperform PSSM400_pKa and other existing TPP predictors.

## Conclusions

In this study, we have conducted empirical comparison and analysis of fourteen existing TPP predictors in terms of multiple perspectives (i.e., feature encoding schemes, feature selection strategies, ML algorithms, evaluation strategies and web server/software usability). We evaluated the existing TPP predictors on the two training and three independent datasets. Our comparative results demonstrated that ThermoPred outperforms other existing TPP predictors in terms of both predictive performance and community utility, while SCMTPP outperforms other existing TPP predictors in terms of high interpretability and simplicity. Although, the existing TPP predictors provide satisfactory prediction performance and promote research progress in this field, there are several issues that need to be addressed. Herein, four crucial aspects for further improving the performance of TPP prediction have been provided as follows: (i) training new models using sequence-to-vector encodings, (ii) using DL-based models, (iii) using an ensemble learning strategy and (vi) developing a web server. We anticipate that this comprehensive review will provide useful insights for researchers in selecting appropriate TPP predictors that are most suitable to deal with their purposes and inspire follow-up research in the future.

## Declaration

### Ethical statement

This review paper does not include animal or human experiments.

### Conflicts of interest

The authors declare no conflict of interest.

### Author contributions statement

WS: Conceptualization, project administration, supervision, investigation, manuscript preparation and revision. PC: Data analysis; data interpretation, investigation and manuscript preparation. NS: Manuscript revision. MMH, MAM and PL: Manuscript preparation. All authors reviewed and approved the manuscript.

### Acknowledgments

This work was fully supported by College of Arts, Media and Technology, Chiang Mai University, and partially supported by Chiang Mai University and Mahidol University. In addition, computational resources were supported by Information Technology Service Center (ITSC) of Chiang Mai University.

## Figures and Tables

**Table 1:**
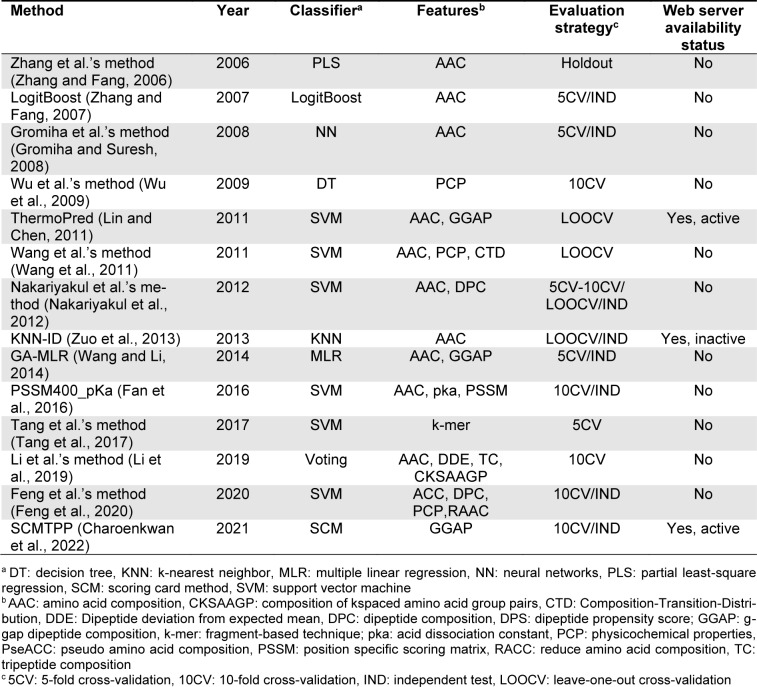
A list of currently available machine learning-based methods for TPP identification summarized in this review

**Table 2:**
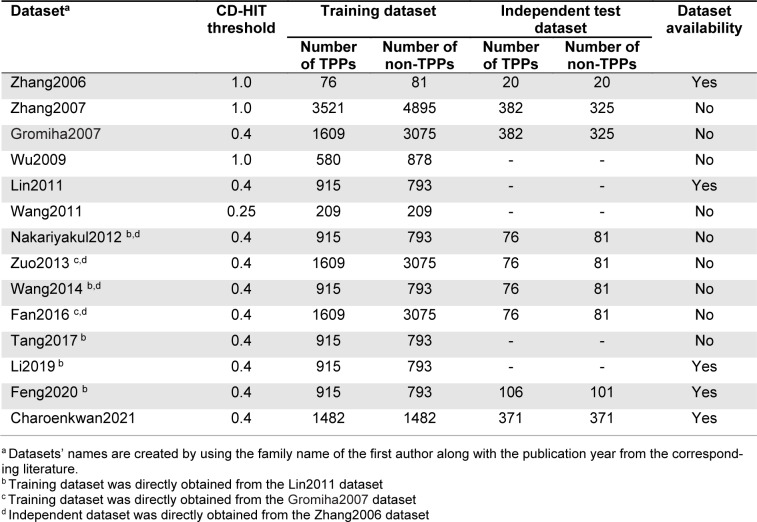
A summary of the training and independent test datasets used for developing the existing TPP predictors

**Table 3:**
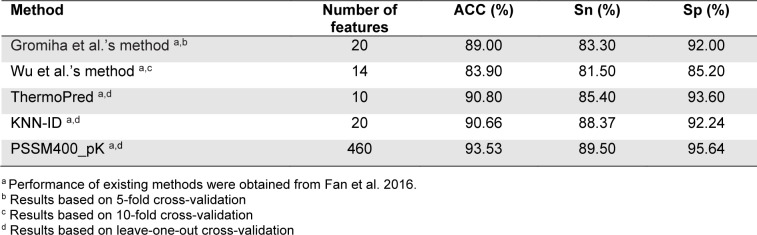
Performance comparison of Gromiha et al.'s method, Wu et al.'s method, ThermoPred, KNN-ID, PSSM400_pK on the Gromiha2007 dataset

**Table 4:**
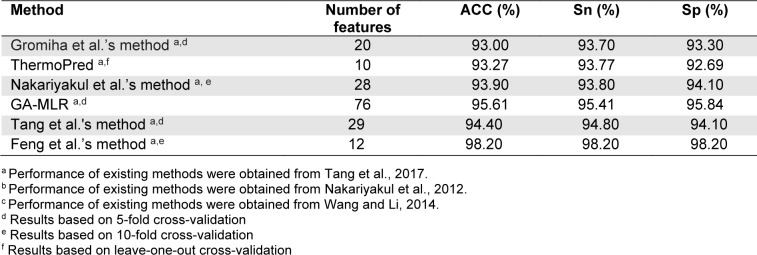
Performance comparison of Gromiha et al.'s method, ThermoPred, Nakariyakul et al.'s method, GA-MLR, Tang et al.'s method and Feng et al.'s method on the Lin2011 dataset

**Table 5:**
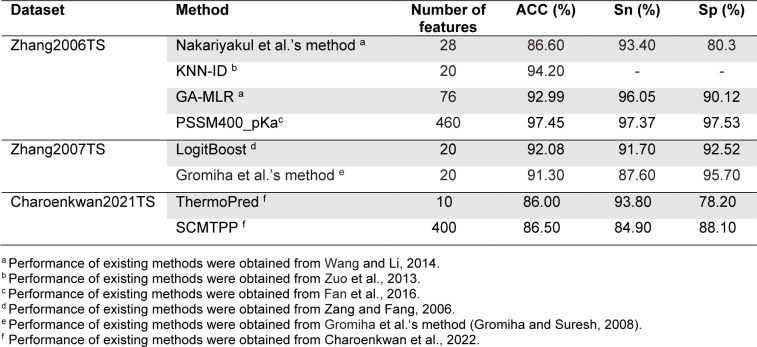
Performance comparison of LogitBoost, Gromiha et al.'s method, ThermoPred and SCMTPP on three independent datasets
